# Coronary artery calcification: concepts and clinical applications

**DOI:** 10.1097/MS9.0000000000002016

**Published:** 2024-04-03

**Authors:** Bing Ji, Xue-Bo Liu

**Affiliations:** Department of Cardiology, Tongji Hospital, School of Medicine, Tongji University, Shanghai, China

**Keywords:** coronary artery calcification, calcified nodules

## Abstract

Vascular calcification is an important hallmark of atherosclerosis. Coronary artery calcification (CAC) implies the presence of coronary artery disease (CAD), irrespective of risk factors or symptoms, is concomitant with the development of advanced atherosclerosis. Coronary thrombosis is the most common clinical end event leading to acute coronary syndrome (ACS). The least common type of pathology associated with thrombosis is the calcified nodule (CN). It usually occurs in elderly patients with severely calcified and tortuous arteries. The prevalence of calcified nodules in patients with ACS may be underestimated due to the lack of easily recognisable diagnostic methods. In this review, the authors will focus on the classification, clinical significance, pathogenesis, and diagnostic evaluation and treatment of CAC to further explore the clinical significance of CN.

In recent years, the presence of calcification in atherosclerosis has been gradually recognised. Vascular calcification is an important hallmark of atherosclerosis irrespective of coronary risk factors or clinical symptoms, and coronary artery calcification (CAC) often accompanies the development of atherosclerosis^1^ and is a major predictor of future adverse cardiovascular events^2,3^. Coronary thrombosis is the most common clinical end event leading to acute coronary syndrome (ACS), and plaque rupture, plaque erosion and calcified nodules (CN) are the three most common pathological mechanisms leading to ACS^4,5^. One of the least common types of pathology associated with thrombosis is CN. It often occurs in elderly patients with severely calcified and tortuous arteries, and the incidence of CN in patients with ACS may be underestimated due to the lack of easily recognisable diagnostic modalities. As the incidence of cardiovascular disease is on the rise with the development of society and changes in lifestyle, the recognition and assessment of CAC have become particularly important.

## Classification of CAC

Calcifications are classified into different types (microcalcifications, extensive dense calcifications, nodular calcifications, and calcified nodules) based on the size and histological appearance of the calcifications under light microscopy^1,6^. Microcalcifications were defined as calcium particles of variable size (0.5 μm ≤ calcification particle diameter 15 μm but 1/4 vessel circumference or peritube length of plaque >3 mm). Nodular calcification is the least common form of CAC and consists of nodular areas of calcification of varying sizes, usually accompanied by a thick and intact fibrous cap, mainly due to the deposition of nodular calcified material in atherosclerotic lesions, where the nodules overlie an intact fibrous cap but the lesion may continue into the mesentery causing disruption of the luminal surface^6,7^. In addition, calcified lesions in the presence of collagen fibres are called collagenous calcifications, whereas calcified lesions without the presence of collagen may show a necrotic core (NC) and are called NC calcifications. Torii *et al*.^7^ reported that fibrous cap rupture was caused by CN, eroded calcified nodules leading to fibrous cap rupture, and lumen-covered thrombus leading to progression of the lesion. Although uncommon, CN can be recognised clinically by intracoronary angiography^8^ and may be associated with a worse clinical prognosis compared to microcalcifications^9^. In addition to the role of microcalcifications and CN in ACS, CAC remains a marker of coronary atherosclerosis (Table 1). The location, density and pressure of calcifications may alter the compliance of the vessel, and calcifications in thin fibrous caps may also alter the tensile forces on the fibrous cap, making the lesion susceptible to rupture. In contrast, microcalcifications (often also referred to as punctate calcifications) are often accompanied by vulnerable plaques. Recent studies have shown that microcalcifications in the fibrous cap may increase local tissue pressure (depending on the distance of one microcalcification point from another and the direction of the microcalcifications in relation to blood flow), leading to plaque instability. In patients treated with statins, calcification without NC can lead to plaque stabilisation^10^.

**TABLE 1:** Modalities to assess coronary artery calcification (CAC)

Diagnostic evaluation	Advantages
Computed tomography (CT)	Measure the density and extent of CAC
Intravascular ultrasound (IVUS)	Provide the distribution and nature of plaques and can detect calcium-enhanced echogenic areas within the vessel wall.
OCT	Allows accurate measurement of plaque thickness
Coronary angiography (CAG)	(High positive predictive value) in detecting CAC
Myocardial perfusion scintigraphy	Non-invasive method to assess and quantify vascular calcification.

OCT, optical coherence tomography.

## Aetiology and mechanisms

Vascular calcification was previously thought to be a benign lesion associated with aging, but it is now increasingly recognised that vascular calcification is a multifactorial-driven pathological process, the result of an imbalance between the promotion and inhibition of mineralisation, driven primarily by vascular smooth muscle cells^11^. The 19th-century pathologist Rudolph Virchow proposed that vascular calcification was the result of the process of ossification^12^. About a century later, Tanimura *et al*.^13^ described a stepwise process of endothelial calcification and pointed out the role of “extracellular matrix vesicles” in endothelial calcification. CAC has been considered a hallmark of coronary atherosclerosis since the 1940s^14,15^. Many local and systemic factors are involved in the formation of CAC, including hyperlipidaemia, inflammation, NC and diabetes. Vascular cells are involved in chondrogenic or osteogenic differentiation, causing mineralisation of membranous bone and the formation of the inner layer of cartilage. Calcified vascular cells are derived from local smooth muscle cells and circulating haematopoietic stem cells (especially in endochondral calcification). There are two forms of vascular calcification---intimal calcification and mesosalpinx calcification, which have different morphological and pathological forms, but they can occur simultaneously^16^. Morphologically, intimal calcification shows punctate and disordered mineral deposits in the inner arterial wall, whereas intimal calcification shows ordered mineral deposits along the elastic lamina of the arterial wall and is associated with chronic kidney disease, diabetes mellitus and advanced age^17–19^. Hypertension and intima-media calcification are closely linked, with the former being a risk factor for promoting calcification and the latter possibly contributing to the development of arterial calcification and hypertension^20–22^. Furthermore, the presence and extent of arterial calcification have been shown to be a major predictor of long-term cardiovascular mortality and adverse cardiovascular events. Intimal calcification is the predominant type of calcification seen in coronary arteries. In intimal calcification, the differentiation of smooth muscle cells (SMCs) to osteoblast-like cells resembles bone formation and is associated with genes such as Bone Morphogenetic Protein 2 (BMP2), Msh Homeobox 2 (MSX2) and alkaline phosphatase (ALP)^23–25^. One of the least common types of pathology associated with thrombosis is CN. The incidence of CN in patients with ACS may be underestimated. Approximately 5% of coronary thrombosis’s are due to calcified nodules protruding into the lumen through a disrupted fibrous cap^26,27^, this vulnerable plaque is usually free of inflammatory cell infiltration^26^ and is often found in severely calcified coronary arteries^26^. The mechanism of formation of CN is unknown. However, a plausible hypothesis is that mechanical stress may fragment the calcium fragments, resulting in small nodules surrounded by fibrin that may eventually erupt through the plaque surface^6^. Bursting CN usually occur in eccentric lesions where their prominence leads to disruption of the overlying endothelial layer, triggering platelet attachment^6^. CN are most commonly found in the right coronary artery (RCA) or left anterior descending (LAD)^26^, often occurring in twisted coronary segments^7^, suggesting that changes in shear stress due to impact and mechanical stresses at the bends of the vessel may lead to the disintegration of the necrotic core calcification into multiple calcified nodules. Torii and colleagues found that when the direction of flow is abruptly changed in a tortuous vessel, the shear forces exerted the greatest effect. Bursting CN should not be confused with “nodular calcification” as the latter is not associated with luminal thrombosis^26^, and although nodular calcification can cause disruption of the mesentery, it rarely continues into the intima^6^. The mechanism of nodular calcification remains unclear, and histology suggests that nodular calcified fibronectin is often present between calcified spicules, along with rare osteoclasts and inflammatory cells, suggesting that at the site of a calcified sheet, fissures and breaks in the area of calcification may be associated with intra-plaque haemorrhage and cellular transformation^4^.

## Diagnostic evaluation

### Computed tomography (CT)

CT has allowed both the density and extent of CAC to be measured objectively (usually calculated using the method of Agatston and colleagues)^28^. Indeed, coronary calcification scores have been widely used to predict the risk of acute coronary events^29^. Although CAC is a hallmark of coronary atheromatous plaque, dense calcification (>400 HU) is usually associated with stable plaque. The characterisation of high-risk plaques has been the subject of intensive research, and new invasive and non-invasive coronary imaging modalities offer new perspectives. CT detection of CAC has been shown to be a more sensitive test than fluoroscopy (90% vs. 52%)^28^. Microcalcifications found in early fibroangiomas, as well as those of SMCs and macrophages, are too small to be identified. However, fibroangiomas containing aggregated calcifications involving large areas of adjacent NC and/or collagen can be observed near the medial edge of the intima on CT, and areas of calcification ranging in size from 1.03 to 1.37 mm^2^ can be detected^30,31^. Recent studies have suggested that the density of calcifications may reflect the overall burden of disease. There is a negative correlation between CAC density measured by CT and the risk of cardiovascular disease (CVD), independent of CAC volume^32^. The Agatston score^28^ has been shown to correlate with histological coronary atheromatous plaque load^33^. CAC score of less than 100 is classified as low risk, 100–399 as intermediate risk and greater than or equal to 400 as high risk. Atherosclerotic plaque load and the occurrence of coronary events are strongly associated^34^, whereas low-risk individuals have a low risk of future coronary events^35^. CAC score only evaluates short-term cardiovascular risk. However, the inconsistency between short-term and long-term risk is well recognised. The CAC score does not take into account the extent and density of coronary artery calcification, which are, however, factors that influence the rate of coronary events^3^. Therefore, more studies are needed to investigate whether the location of CAC lesions and calcification patterns are important predictors of cardiovascular events.

### Intravascular ultrasound (IVUS)

IVUS involves placing a catheter containing a high-frequency ultrasound transducer into the lumen of a coronary artery for cross-sectional imaging. As such, IVUS provides a more comprehensive view of the distribution and nature of plaques than coronary angiography (CAG) and can detect calcium-enhanced echogenic areas within the vessel wall. Intensely echogenic areas within the vessel wall. IVUS has been shown to have high sensitivity (90%) and specificity (100%)^36^. IVUS is not only more sensitive than CAG in identifying CAC, but also provides information about the morphology of the calcification (curvature, length, superficial/deep). CN are fibrous cap disruptions detected on prominent calcified plaques^8^. CN are convex in the lumen with an irregular surface^37^ and are mainly classified as intimal calcification, basement membrane calcification and mixed calcification. Lesions with CN tend to have extensive calcium patches, but in IVUS, calcified plaques have artefacts preventing measurement of their thickness. Although CAG can only provide a semi-quantitative assessment of calcification (absent, moderate, severe), the PROSPECT study identified predictors of coronary plaque complications such as thin fibrous cap atherosclerotic plaques, lumen area, plaque loading and dense calcification^38^. Previous quantitative methods for calcification have focused on the measurement of calcium arcs at the lesion site^39^, while the measurement of calcification at other sites seems to have been neglected. The Calcification Index is a three-dimensional IVUS measurement designed to determine the total amount of calcification throughout the vessel. It is calculated as total calcification length/lumen length × maximum calcification arc/360◦. The arc of calcification is measured using an electronic protractor in electronic software, and the calcification length can be measured by a motorised sensor pull-back system^40^. Researchers have found that the calcification index is highly consistent with the results of histopathological calcification assessment^41^. Calcification index calculation is useful for assessing calcification progression in clinical studies, especially before and after interventions, as a baseline and follow-up outcome^30,31,42^.

### Optical coherence tomography (OCT)

OCT has a higher resolution and can penetrate coronary artery calcium. OCT has shown greater accuracy in diagnosing calcification in histological validation studies with high sensitivity and specificity compared to IVUS. OCT with 10-fold spatial resolution allows further analysis of calcium deposits in the coronary artery wall. Calcification deposits can be seen as well-defined low-intensity, low-attenuation, homogeneous low-signal areas on OCT, which allows accurate measurement of plaque thickness^43,44^. At the lesion site, three morphologies of calcification subtypes were detected by OCT: eruptive CN, superficial calcifications and calcified protrusions^45^. Most of the CN had superficial macroscopic calcifications. Exploding CN present as CN protruding into the lumen by disrupting the fibrous cap, similar to histopathologically CN^44^, and like lipid-rich thin fibrous cap lesions, 83.3% of exploding CN are primarily associated with an overlying red thrombus^44^. OCT is advantageous for the detection of thrombus, demarcation of superficial and deep boundaries of the calcification, and plaques in exploding calcified nodules. However, OCT has some limitations; in ACS offender lesions, a large number of thrombi may lead to strong attenuation of near-infrared light, interfering with the accurate assessment of plaque morphology by OCT. Detection of calcification if the border is not clear enough to distinguish between lipid core and calcification, or in the presence of an overlying thrombus, can be better achieved by IVUS and virtual histology-IVUS^46^. Recently, Kobayashi et al. reported their results of OCT in patients with ACS, which showed that calcified nodules were the least common cause of ACS (6%)^47,48^. In addition, massive calcification was significantly associated with higher mortality. Furthermore, massive calcification was significantly associated with higher mortality, myocardial infarction, and target-vessel revascularisation after percutaneous coronary intervention (PCI) compared to other causes of ACS (i.e., plaque rupture and erosion). Another OCT study conducted by Lee et al. demonstrated a cause-and-effect relationship between the location of CN and the degree of angiographic vasodilatation of the coronary arteries. CN were seen in 4.2% of 889 cases (48% of ACS cases) and were mostly located in the intima-media of coronary arteries with large coronary hinge motion and in the mid-right coronary artery, suggesting that mechanical disruption of lamellar calcification due to coronary artery motion may be one of the mechanisms of calcified nodule formation^49^. The incidence of CN was reported to be 5% in a study of 442 autopsy cases of sudden coronary death.CN occur almost exclusively in older adults with highly calcified and tortuous arteries. The histological features are sheet-like calcification^50^.

### Coronary angiography

CAG has a low to moderate sensitivity but high specificity (high positive predictive value) in detecting CAC compared to IVUS and CT^28,51^. Angiographic CAC is usually classified into three groups: absent/mild, moderate and severe. Severe. Severe calcification is usually defined as radiological calcification where no cardiac motion is seen prior to contrast injection. Usually affecting both sides of the arterial lumen, moderate calcification is defined as radiolucency found only during the cardiac cycle prior to contrast injection. Moderate calcification is defined as radiolucent spots found only during the cardiac cycle before contrast injection^52^.

### Myocardial perfusion scintigraphy

Although IVUS is an effective tool for the study of atherosclerotic calcification, with a sensitivity of 100% and specificity of 99%^53^, its invasive use is limited to the secondary prevention population. In clinical practice, the most effective screening method for assessing atherosclerosis remains the Calcification Score, which reclassifies patients at intermediate to high cardiovascular risk. Functional tests for ischaemia assessment, such as myocardial perfusion scintigraphy or stress echocardiography, are currently recommended for patients with an anatomical atherosclerotic Agatston’s Calcification Score greater than 400^54^. The tracer 18F-NaF is highly specific and sensitive, and by detecting 18F-NaF within the arterial wall, PET can provide a non-invasive method to assess and quantify vascular calcification. By detecting 18F-NaF within the arterial wall, 18F-NaF PET can provide a non-invasive method to assess and quantify vascular calcification^55^. Due to its lack of anatomical detail, PET scanners are often used in conjunction with CT or MRI scanners to take full advantage of high-sensitivity molecular imaging and high-resolution anatomical imaging. 18F-NaF uptake has been shown to correlate with 10-year atherosclerotic cardiovascular disease risk scores^56^. It has been further demonstrated that 18F-NaF PET helps to improve risk stratification of patients with suspected coronary artery disease (CAD). A recent study confirmed that better risk stratification can be achieved by combining 18F-NaF PE with quantitative plaque analysis and clinical information obtained by machine^57^. In a study including 34 patients with malignant tumours or orthopaedic diseases, researchers compared baseline PET imaging results with CT images after ~1 year of follow-up The results of baseline PET imaging results were compared with CT images after ~1 year of follow-up, and the results showed that 18F-NaF uptake has the potential to predict the progression of calcification scores, which have been reported to be a strong predictor of future cardiovascular risk^56^. A prospective observational study reported that patients with CAD are known to have a more than sevenfold increased risk of fatal or non-fatal myocardial infarction^57^. However, data on the dynamics of 18F-NaF uptake during the progression of atherosclerosis are lacking to date. Theoretically, the simultaneous application of 18F-NaF and 18F-fluorodeoxyglucose (18F-FDG) probes has the potential to facilitate dual assessment. 18F-FDG probes may be useful for dual assessment of plaque microcalcification and inflammatory activity. Dual assessment of plaque microcalcification and inflammatory activity, thus providing a complementary assessment of atherosclerosis. A recent study included 26 patients with acute ischaemic stroke and examined the possible role of dual tracer imaging in atherosclerosis^58^.

## Interventional treatment

CAC poses a considerable challenge to PCI. A significant amount of CAC limits the effectiveness of balloon angioplasty and hinders stent delivery and stent expansion^59^. To address these issues, calcium modification techniques such as high-pressure, scored or cut balloon angioplasty or atherosclerotic intimal spinning are commonly used with variable results. Regardless of the technique used, PCI of heavily calcified offender vessels may increase the risk of no-reflow, coronary artery entrapment, stent damage or stent dislodgement, and periprocedural myocardial infarction (MI)^60^.

### Drug-eluting stents (DES)

DES have a substantially lower rate of in-stent restenosis compared with bare-metal stents^61^, and the superiority of first-generation DES over bare-metal stents has been demonstrated in a variety of subgroups of patients, including those with complex calcified lesions^61,62^. However, because first-generation DES increase the risk of stent thrombosis, subsequent iterations of DES have been designed to reduce this risk. With improvements in antiproliferative drugs, polymers, thin stent frames, and clinical performance, second-generation DES have replaced their predecessors in modern practice^61^. However, it remains unclear whether these second-generation DES improve PCI outcomes in patients with severe CAC. A previous meta-analysis by Nishida and colleagues^5^ showed that second-generation DES were associated with a lower target lesion failure (TLF) rate in PCI treatment of severely calcified lesions compared with first-generation DES. However, this correlation did not reach statistical significance and therefore no reliable conclusions could be drawn^63^. Guedeney *et al*.^63^ conducted a meta-analysis of 18 randomised controlled trials to assess the outcome and rainy day of second-generation DES for PCI in the presence of significant CAC. Of the 19 833 patients included, moderate/severe CAC was confirmed in greater than or equal to 1 target lesion in 6211 (31.3%) patients. Nearly 60% of patients in both groups (no/mild versus moderate/severe CAC) received second-generation DES. Study endpoints included a patient-oriented composite endpoint (POCE) (including death, myocardial infarction, or any haemodilution) and a composite endpoint of target lesion failure rate [TLF, defined as cardiac death, target-vessel myocardial infarction, or ischaemia-driven target lesion haemodilution (ID-TLR)]. Patients with moderate/severe CAC were older and more likely to have had coronary artery bypass grafting compared with patients with no/mild CAC. In addition, patients with C-shaped lesions, left anterior descending end-vessels, and Thrombolysis in Myocardial Infarction (TIMI) flow grades 0–1 were more common in the moderate/severe CAC group, had longer total stent lengths, and had more frequent multivessel PCI. Patients with moderate/severe CAC had a higher incidence of both study endpoints (POCE and TLF) (30.5% vs. 25.4%; *P* < 0.001 and 16.4% vs. 12.0%; *P* < 0.001, respectively). After adjustment for patient baseline and surgical characteristics, moderate/severe CAC was associated with a higher 5-year risk of POCE [adjusted hazard ratio (aHR): 1.12; 95% CI: 1.05–1.20)], TLF (aHR: 1.21; 95% CI: 1.09–1.34), and individual endpoints of death, myocardial infarction, and ID-TLR were associated. When stratified by stent type, PCI with second-generation DES was associated with reductions in 5-year POCE (aHR: 0.88; 95% CI: 0.78–1.00) and TLF (aHR: 0.73; 95% CI: 0.61–0.87) as well as the individual endpoints of death, MI, ID-TLR, and stent thrombosis.0–1-year and 1–5-year Marker analyses confirmed the benefits of second-generation DES in terms of POCE, TLF, and stent thrombosis. Several studies have shown that DES are more effective than bare-metal stents (BMS) in calcified lesions. DES showed less neointimal hyperplasia in calcified (and non-calcified) lesions compared to BMS, reducing late angiographic loss, restenosis and repeat revascularisation^64^. It has been shown that in calcified lesions, DES and BMS have similar rates of stent thrombosis and comparable rates of death and myocardial infarction^64^.

### Modified balloon

Modified balloons are a useful adjunct to help adequately pre-dilate moderately calcified lesions to achieve greater lumen expansion than conventional balloon angioplasty^65^. The surface of the cutting balloon is longitudinally fitted with microblades, and the coronary cutting balloon is 2.0–4.0 mm in diameter. In contrast, the surface of the scoring balloon is transversely mounted with microblades and its scoring element runs along the surface of the balloon. The AngioSculpt RX (Philips) scoring balloon consists of a semi-compliant balloon and three nitinol helical scribes, which has a lower profile than the cutting balloon, and its size ranges from 2 to 3.5 mm. Both can improve vascular compliance by creating discrete incisions in the plaque, and both can improve vascular compliance by creating discontinuous incisions in the plaque that enlarge the lesion. In addition, they reduce elastic recoil, prevent uncontrolled stripping, and are used in stented lesions that restenosis after dilatation^65,66^. The largest published registry study showed that the largest registry experience of Angio-Sculpt for the treatment of 745 lesions with at least moderate calcification reported a 30-day major adverse cardiac event (MACE) of 2.9% and a procedure success rate of 97.9%^67^. Their use is limited to lesions with severe calcification and those that cannot be traversed by a standard pre-dilated balloon. Previous data studies have shown that indentation balloons and ultrahigh-pressure PTCA balloons (OPN balloons) appear to be equally safe, but it has also been shown that surgical success is more likely with OPN balloons^32^, and that surgical success rates are higher with OPN balloons^68^.

### Endovascular lithotripsy

Endovascular lithotripsy provides circumferential pulsatile mechanical energy through an intracoronary balloon that selectively destroys superficial and deep layers of calcium in the vessel wall^69^. Balloon diameters range from 2.5 to 4.0 mm, with a 1:1 balloon-to-vessel-wall dimension, and each balloon emits eight pulses for 10 seconds. Intravenous luminal calcium introduction is most effectively achieved by fracturing the more burdensome peripheral calcific arcs, and no pre-dilatation is required after IVL is performed. In the DISRUPT CAD III trial, 431 patients with severe calcified lesions were treated with lithotripsy, with a mean calcification length of 47.9 mm, a mean calcification angle of 292°, and a thickness of 0.96 mm. Procedural success was defined as successful stent implantation and angiographic detection of less than 50% residual stenosis. The procedural success rate was 92.4%, and the MACE-free rate at 30 days was 92.2%^70^. The CVX-300 Philips is a xenon-chlorine laser capable of emitting 30 to 80 mJ/mm^2^ of energy at a rate of 25–80 pulses per second, with 25–80 pulses emitted per second, resulting in molecular bonding, apoptosis, and the expansion and explosion of gas bubbles in liquid media. In calcified lesions, these mechanisms lead to changes in plaque compliance at depths of up to 1 mm^65^. ELCA consists of a 0.9-mm-diameter catheter passed through a 0.014-inch guidewire, with the wire placed distal to the lesion, and then the laser is moved until it is in direct contact with the lesion, and, for maximal effect, the laser is advanced at a rate of 1 mm/s. ECLA is indicated in cases where a balloon cannot be dilated lesions, as well as insufficient stent dilation and proximal chronic total occlusion lesions^67^. Saline is less able to absorb energy than blood or contrast media. Saline is less able to absorb energy than blood or contrast media and has, therefore, become standard practice to reduce the risk of entrapment and perforation. Infusion of saline is less able to absorb energy than blood or contrast media and has therefore become standard practice to reduce the risk of dissection and perforation^67,70^. However, saline has limited efficacy in severely calcified lesions. Therefore, its use is limited to situations where a rotating guidewire or microcatheter cannot be passed through the lesion. The underlying plaque is altered with ELCA, followed by balloon dilatation using a non-compliant balloon. Balloon dilatation should be performed using a non-compliant balloon sized 1:1 to the lumen diameter to ensure adequate dilatation of the lesion.

### Drug therapy 5.1 Statins

Some clinical studies have shown that intensive lipid-lowering therapy reduces the risk of cardiovascular events but promotes the progression of CAC^71^. A meta-analysis of eight clinical studies has shown that intensive therapy with statins reduces plaque volume but increases the progression of CAC^72^. These studies suggest that plaques with dense calcification are more stable and less likely to rupture. The mechanism by which statins induce plaque stabilisation in the presence of active calcification is unknown and remains a direction for further research in the future. Researchers have speculated that statins may lead to localised vitamin K2 deficiency in vascular endothelial cells, thereby impairing the inhibitory effect of vitamin K on vascular calcification^73^. In the St Francis study, 1005 patients with calcification scores greater than or equal to 80th percentile for age and sex were randomised to atorvastatin 20 mg/day or placebo^74.^ The results demonstrated that atorvastatin lowered LDL levels and significantly reduced MACE events, but had no effect on the progression of CAC. Although selective inhibition of the renin-angiotensin-aldosterone system but not a significant reduction in CAC has not yet been investigated to show whether regulation of the nuclear factor-γ receptor activator or proliferation-activated receptor gamma pathway affects CAC in humans^75^.

### Warfarin

Andrews *et al*.^76^ showed that warfarin treatment in patients with coronary artery disease was associated with increased progression of CAC. This study was a patient-level post-hoc analysis of eight prospective randomised clinical trials using IVUS on matched arterial segments in patients with coronary artery disease treated with warfarin (*n* = 171) or not treated with warfarin (n = 4,129).Andrews *et al*.^76^ derived a reliable correlation between warfarin from data obtained from eight different consecutive clinical trials, and reliably concluded that warfarin use was associated with changes in CAC. Warfarin use was not associated with CAC progression and baseline CAC, atheromatous plaque volume, concomitant statin therapy, or renal function. This study is consistent with cross-sectional clinical studies reporting a promoting effect of warfarin on arterial calcification^77^. But is the accelerated progression of CAC observed in patients treated with warfarin a good or bad thing? Warfarin is a vitamin K antagonist that targets both vitamin K-dependent coagulation factors and vitamin K-dependent extrahepatic proteins such as vascular smooth muscle cell-derived matrix Gla protein (MGP). The important function of vascular MGP in inhibiting arterial calcification has been demonstrated in MGP knockout mice and in experimental animals treated with warfarin; in both groups of animals, the onset and progression of vascular calcification were induced by MGP^78^. Animal experiments also showed that warfarin increased the intimal calcification of atherosclerotic plaques but did not affect the volume of atherosclerotic plaques^79^. The same animal study also showed that warfarin treatment promoted intimal microcalcification, increased apoptosis and facilitated outward remodelling, shifting atherosclerotic plaques towards a vulnerable phenotype. Further clinical studies are needed to assess the impact of long-term warfarin use on clinical events in patients with coronary artery disease. Such studies will also explore the true value of CAC as a biomarker of adverse cardiovascular events.

## Summary

Pathological and imaging data together emphasise that “not all calcifications are equal” when assessing the role of calcification in cardiovascular disease. The formation of CAC is a pathological process driven by multiple factors and is one of the manifestations of atherosclerosis. Better identification of coronary calcification can be achieved by diagnostic evaluations such as CT, IVUS, and OCT; however, there is a need for further improvement in the future in the identification of CN, and the early detection of patients with intermediate to high cardiovascular risk for clinical classification. It is possible that the effect of plaque stabilisation by statins might be partially explained by enhancement of calcification in the plaque necrotic core, although the basic mechanism is still unclear. Further investigation is required.

## Ethics approval and consent to participate

The committee assessed the planned project as ethically unobjectionable. Written consent to participate were obtained from all study participants.

## Patient consent for publication

Written informed consent was obtained from a legally authorised representative(s) for anonymized patient information to be published in this article. The manuscript does not contain any individual person’s data in any form.

## Source of funding

Not applicable.

## Contribution

All authors have contributed significantly and that all authors agree with the content of the manuscript.

## Conflicts of interest disclosure

The authors have no conflict of interests to declare.

## Research registration unique identifying number (UIN)

Not applicable.

## Guarantor

Not applicable.

## Availability of data and materials

All data generated or analysed during this study are included in this published article.
